# Evidence for a High Temperature Whisker Growth Mechanism Active in Tungsten during In Situ Nanopillar Compression

**DOI:** 10.3390/nano11092429

**Published:** 2021-09-18

**Authors:** Gowtham Sriram Jawaharram, Christopher M. Barr, Khalid Hattar, Shen J. Dillon

**Affiliations:** 1 Department of Materials Science and Engineering, University of Illinois Urbana-Champaign, Urbana, IL 61801, USA; gowtham.first@gmail.com; 2 Sandia National Laboratories, P.O. Box 5800-1056, Albuquerque, NM 87185, USA; cmbarr20@gmail.com (C.M.B.); khattar@sandia.gov (K.H.); 3 Department of Materials Science and Engineering, University of California, Irvine, CA 92697, USA

**Keywords:** tungsten, fuzz, whisker, in situ, transmission electron microscopy

## Abstract

A series of nanopillar compression tests were performed on tungsten as a function of temperature using in situ transmission electron microscopy with localized laser heating. Surface oxidation was observed to form on the pillars and grow in thickness with increasing temperature. Deformation between 850 °C and 1120 °C is facilitated by long-range diffusional transport from the tungsten pillar onto adjacent regions of the Y_2_O_3_-stabilized ZrO_2_ indenter. The constraint imposed by the surface oxidation is hypothesized to underly this mechanism for localized plasticity, which is generally the so-called whisker growth mechanism. The results are discussed in context of the tungsten fuzz growth mechanism in He plasma-facing environments. The two processes exhibit similar morphological features and the conditions under which fuzz evolves appear to satisfy the conditions necessary to induce whisker growth.

## 1. Introduction

Tungsten has been considered as a helium (He) plasma-facing material for fusion reactors due to its high physical sputtering threshold energy, stability against hydride formation, high melting temperature, good thermal conductivity, and low vapor pressure [1,2,3]. Any vaporized or eroded heavy elements entering the plasma in a fusion reactor can degrade performance, meaning that prevention of erosion and evaporation of the plasma-facing material is a critical materials design consideration. Therefore, understanding structure and chemistry of the plasma-facing surface, as well as its evolution, is of potential importance. Tungsten is prone to forming so-called ‘fuzz’ at high temperatures when interacting with He particle fluxes inherent to the reactor wall environment. The fuzz consists of tungsten metal filaments with widths on the order of nanometers that can grow to lengths on the micron to millimeter scale depending on the environmental conditions that produced them [4,5]. These features form between approximately 700 °C and 1700 °C and when low energy ions impinge the surface, typically greater than ≈20 eV [2]. Existing models for tungsten fuzz evolution assume that the formation of He bubbles drives a mechanical response that produces the fuzz morphology. This has been discussed, for example, in terms of a viscoelastic response [6] or a loop-punching and bubble burst mechanism [7]. It has also been suggested that surface diffusion may play an important role in evolving fuzz from what would alternatively form nanoporous structures at lower temperatures [8].

Various proposed mechanisms for fuzz growth invoke aspects of deformation, creep, and irradiation-induced creep of nanostructured tungsten at high temperatures [6,7,8,9]. High temperature mechanical properties and the deformation creep response of macroscopic tungsten samples have been thoroughly investigated within the literature, and provide for definition of deformation mechanism maps for coarse-grained samples [10,11]. It has been observed that tungsten fuzz growth can proceed after ion irradiation ceases, which suggests a time dependent response that is consistent with a creep mechanism [8]. The mechanical properties of nanoscale pillars, whiskers, and filaments are well known to differ significantly from their bulk counterparts particularly at room temperature where most measurements have been performed [12,13,14,15]. The yield strength of W pillars, for example, increase by a factor of ≈5 upon reducing pillar dimension from ≈900 nm to ≈200 nm [13]. Nanoscale particles and filaments are also subject to mechanical response influenced by capillary effects due to the relatively high-volume fraction of interfaces [16,17]. Unresolved questions, therefore, remain regarding whether nanoscale size effects influence the predicted mechanical properties of tungsten at high temperatures and timescales where bulk and or interfacial diffusion are facile and what the associated deformation modes are.

The mechanical properties of nanoscale specimen have been evaluated via a variety of techniques over the last several decades [18,19,20]. Few techniques, however, are well suited to high temperature mechanical testing. Commercial nanomechanical testers have been developed with heating stages that can access temperatures in the range of 600 °C to 800 °C, which typically employ Joule heating [21]. The temperature regime relevant to large-scale tungsten fuzz growth lies at or above this range. An alternative approach combining laser heating and nanomechanical testing has recently been developed, which enables access to temperature in excess of 2500 °C in ZrO_2_-based samples [22]. The approach utilizes a laser spot that is large relative to the length scale of the specimen, which supports temperature uniformity of the sample, but is small on the length scale of the overall mechanical tester, while also avoiding heating of the electronic components of the tester. Comparable high temperatures may be challenging to achieve in many bulk alloys due to a combination of sample reflectivity and high thermal conductivity. Testing thin alloy films on thermally insulating substrates, however, could sufficiently suppress thermal conduction to enable ultrahigh temperature experiments.

A primary goal of this work is to understand whether the high temperature deformation response of small-scale features underlies the fuzz growth process. The work measures the mechanical response of tungsten nanopillars as a function of temperature between room temperature and approximately 1250 °C. Above about 850 °C, a significant diffusional creep component of yielding is observed in the nanopillars. Facile diffusional mediated stress relaxation mechanisms are, therefore, active in the temperature range and length scales relevant to tungsten fuzz formation. Furthermore, the existence of a surface oxidation is shown to strongly influence the deformation mode in the regime where diffusional creep is facile.

## 2. Materials and Methods

Tungsten films were prepared via vapor deposition onto Y_2_O_3_-stabilized ZrO_2_ single crystal substrates. The substrates were initially prepared by mechanically polishing the reverse side of the substrate until the total sample was of thickness on the order of 100 μm. Tungsten films were grown to a thickness of ~1 μm on the pre-polished side of the substrate using DC magnetron sputtering from a high purity (>99.95%) target in a system with a base pressure of ~5 × 10^−8^ torr and under an Ar pressure of ~3 × 10^−3^ torr. Approximately square cross-section pillars were prepared using focused ion beam milling (FIB, FEI Helios). Minor gallium (Ga) contamination is typical of such preparation. The effects of Ga impurities on the mechanical properties and creep rates of W are not well known. The focus of this work, however, is to observe how deformation mechanisms are affected by temperature. Pillars were milled in two orthoganol directions to avoid tapering of the pillar geometry. The average cross-sectional area of the pillars and associated standard deviation were 9.1 × 10^4^ nm^2^ 3.5 × 10^4^ nm^2^.

Microscale diamond indenter tips were found to be geometrically unstable at temperatures of interest in this work. As an alternative, a single crystal Y_2_O_3_ stabilized ZrO_2_ indenter was prepared using a combination of mechanical polishing and focused ion beam milling. The final indenter was on the order of 10 μm in diameter. The base of the indenter was coated with a thin layer of Au-Pd and a shadow mask was used to prevent coating of the tip. The goal of this preparation is to suppress electrostatic charging, while avoiding the formation of surface deposits on the tip. Preparing the indenter and sample from the same material and of similar geometry is employed as a strategy to promote temperature uniformity between the sample and indenter. This indenter was mounted on a Bruker PI-95 Picoindenter. The sample was tested in a JEOL 2100 TEM outfitted with a 20 W 1056 nm IR laser aligned parallel to the electron beam with a spot size of ~50 μm. Indentations were performed under displacement control at a rate of 5 nm s^−1^. Samples were tested sequencially at increasing temperatures, except the sample tested at 850 °C, which was tested after the sample at 1250 °C that reacted during compression. This reaction suggested that higher temperature experiments were not practically feasible, and thus the remaining pillar was tested at a lower temperature. Samples were characterized ex situ after compression using scanning electron microscopy (SEM). Pillars were observed ex situ in the SEM to ensure pillars did not bend out of plane and relativel deformed uniformly along the axes parallel and perpendicular to the direction of observation in the in situ experiment.

The approximate local temperature of the pillars was determined from electron diffraction patterns and the thermal expansion coefficient following the method developed by Niekiel et al. [23]. A custom Python code was used for this procedure. The determination of the lattice constant involves first determining the accurate diffraction pattern center. An initial center was first guessed and passed as input to the code. The diffraction rings were then identified based on morphological image operations “dilation” and “erosion”. This method ensures all diffraction spots, above a minimum threshold value, are identified. The identified spots from the various rings are normalized to a certain radius away from the center. These identified spots are then used to optimize the initially presumed diffraction pattern center using a least square regression. Once the optimum center has been identified, the remaining distortions are a result of lens aberrations in the TEM. An elliptical distortion equation is as follows:

(1) $$\left(r,\varphi \right)=r\frac{1-\eta^{2}}{\sqrt{1+\eta^{2}-2\eta \cos\left(n\left(\varphi +\omega \right)\right)}}$$

where, *η* is the distortion factor given by (1−rminrmax1+rminrmax), *ω* is the orientation of the major axis, and *n* describes the order of the aberrations, *n* = 2, 3, 4 corresponds to two, three, and four fold distortions, respectively.

## 3. Results

**Figure 1:**
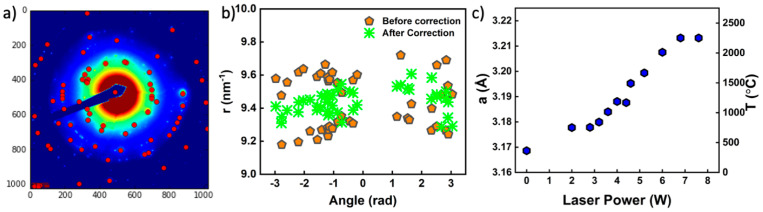
(**a**) Example colorized electron diffraction pattern obtained from tungsten showing He location of diffraction spots identified by a custom python code. (**b**) Uncorrected and corrected positions of diffraction spots associated with a set of reflections. (**c**) Plot of the calculated lattice parameter as a function of applied laser power.

Figure 1 shows an example diffraction pattern, a plot of corrected and uncorrected diffraction peak positions, and a plot of the lattice parameter versus laser power. For temperatures in excess of T ≈ 1250 °C, the ring pattern in the diffraction was lost and slowly evolved to a single crystal pattern at the highest temperatures. The loss of the ring pattern degrades the efficacy of the calibration method, thus the temperature values are less reliable above this range. The samples were, nevertheless, only mechanically tested up to T = 1250 °C. The higher temperature diffraction data is included in Figure 1 primarily to highlight that the experimental method could be extended to higher temperatures in other metal systems under appropriate vacuum conditions. The lattice parameter expansion is, nevertheless, reasonably linear with applied laser power above the threshold value as anticipated from prior work [24,25]. The primary diffraction peaks correspond to body-centered cubic tungsten up to approximately 2600 °C [26]. The diffraction patterns are obtained from a larger area surrounding the pillar after mechanical testing. Past experiments performed in this microscope indicate that oxidation of Ta becomes facile at ≈700 °C [27] and oxidation of NiCoFeCrMn becomes facile at ≈800 °C [25]. As discussed below, a thin oxidation scale is observed on samples at lower temperatures, which is anticipated to become thermodynamically unstable under vacuum within the microscope above approximately 1500–1600 °C, depending on the oxygen concentration in the vacuum. Under ambient conditions, tungsten oxides evaporate at an appreciable rate on the length scale of our experiments at temperatures exceeding 1200 °C [28]. If oxidation were appreciable at these temperatures, then the nanoscale specimen would evaporate rapidly. It is, therefore, concluded that samples primarily remain metallic despite the observation of thicker oxidation products between ≈850 °C and ≈1250 °C. The atmosphere within the TEM is difficult to measure explicitly; nevertheless, it is anticipated that some oxidation should occur at lower temperatures and that the tungsten oxide become unstable to evaporation at 1450 °C and reduction in vacuum at comparable temperatures [29]. The role of the electron beam in affecting the oxide is not generally clear; it can both ionize gaseous species in the vacuum, enhancing the driving force for oxidation, and can also destabilize the oxidation products through ionization and displacement damage [30]. The latter process should, however, dominate as the vacuum pressure decreases. Above ≈1250 °C, the nanopillars are not geometrically stable, which limits the maximum testing temperature for nanopillar compression experiments.

**Figure 2:**
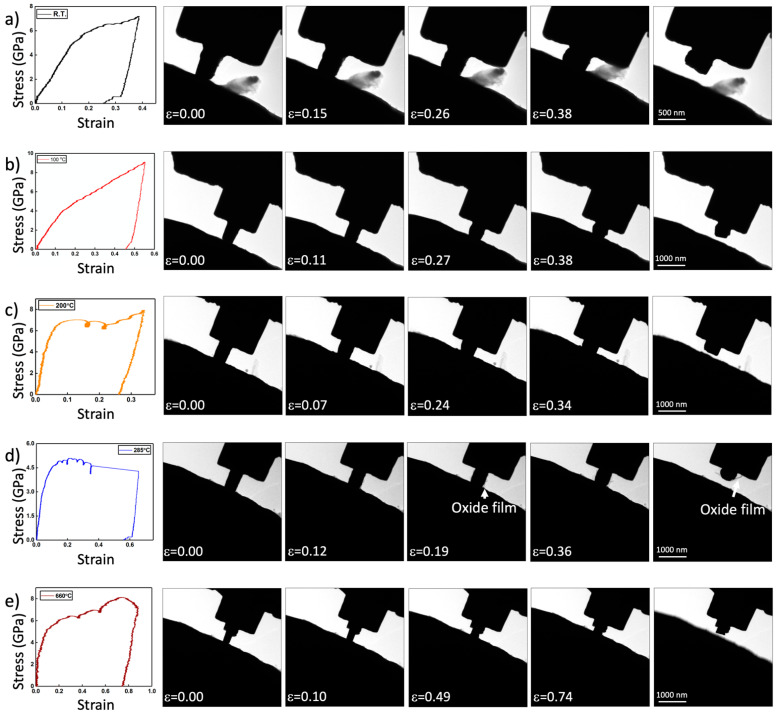
A series of nanopillar compression experiments performed at (**a**) room temperature, (**b**) 100 °C, (**c**) 200 °C, (**d**) 285 °C, and (**e**) 660 °C. including time-lapse images and stress–strain curves. Of note is the observation of a thin scale debonding from the tungsten pillar while testing at 285 °C.

Figure 2 depicts example time-lapse images of pillar compressions and associated load-displacement curves for samples tested at room temperature, 100 °C, 200 °C, 285 °C, and 660 °C. In this temperature range, all measured yield strengths are greater than ≈4.5 GPa. Nanocrystalline tungsten thin films exhibit a hardness between 15 and 20 GPa [31,32], which is reasonably in line with our measurements considering that hardness in nanograin materials typically exceeds yield strength by a factor of ≈2.5–4 [33]. The yield strength is weakly temperature dependent in this range, if at all. The samples are all nanocrystalline at and below 285 °C. At 285 °C, an approximately 10–20-nm-thick film appears to fracture away from the pillar surface during compression. This film is amorphous and is anticipated be associated with the decohesion of an oxidation product on the pillar.

**Figure 3:**
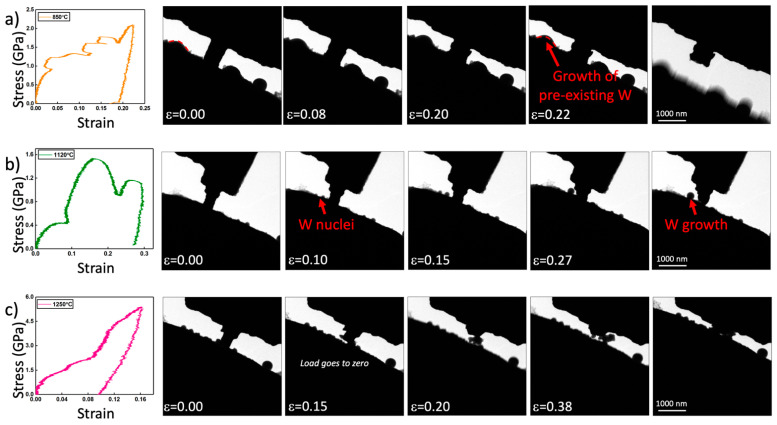
A series of nanopillar compression experiments performed at different temperatures between 850 °C and 1250 °C including time-lapse images and stress–strain curves. At 850 °C, shear is observed in the pillar along with some growth of tungsten particles that pre-existed on the surface of the YSZ indenter. Experiments performed at 850 °C demonstrate the nucleation and growth of tungsten particles on the YSZ indenter as the pillar is compressed. At 1250 °C, the pillar undergoes initial loading but begins reacting rapidly. The load returns to zero as the pillar continues to react. The samples in this figure were tested sequencially (**b**), followed by (**c**) and then (**a**), which accounts for W particles existing on the indenter surface prior to loading.

Figure 3 shows pillars loaded in compression at 850 °C, 1120 °C, and 1250 °C. At 850 °C, the yield strength begins to decrease rapidly. Deformation of the pillar is more inhomogeneous exhibiting localized plasticity (see Figure 3a). This is consistent with anticipated coarsening of the nanograined tungsten via grain growth as observed by a significant reduction in diffraction spots. Grain growth in tungsten films has been shown to becomes facile above 650 °C and should grow to the lengthscale of the pillar diameter by that temperature [34]. The observation of shear steps at the surface, thus, suggest that the grain diameters have approached the width of the pillar by 850 °C. The reduction in strength at this temperature may be attributed in part to the loss of nanograin strengthening via a Hall–Petch-type mechanism. This conjecture is supported, in part, by measurements performed at lower temperatures after pre-annealing, indicating a considerable reduction in yield strength relative to the as-deposited materials tested at room temperature.

At 1120 °C, a rather unusual deformation mode is apparent as outlined in Figure 3b. As the pillar is compressed, several round particles nucleate and grow on the ZrO_2_ indenter adjacent to the indenter. These particles appear to coarsen into a single large particle via Ostwald ripening. The load vs. displacement curve displays several large oscillations in load associated with a plastic response in the sample. These oscillations temporally correlate with the nucleation and growth of the truncated spherical particles growing on the surface. After compression, the pillar appears to have reduced in volume, to the extent that this can be approximated from the two-dimensional image. Measurements of high temperature deformation in ZrO_2_, i.e., the indenter, suggest that, at such stresses and temperature, no plastic deformation is anticipated. As a result, the growth of particles on the ZrO_2_ is associated with the plastic response of the tungsten pillars. This is futher supported by conservation of volume arguments, wherein the reduction in pillar volume is sufficiently large that it must be accounted for by mass transport away. What remains unclear from these results is why the transport of mass from the pillar is de-localized away from pillar itself. For creep, a flux is generally anticipated from regions of high compressive stress to relatively tensile or stress free regions, such as a free surface. Calculations of Coble creep strain rate at 1120 °C, based on values from the literature and on length scales of the pillar radius [11], predict a value on the order of 10^−1^. This supports the hypothesis that Coble creep can be active in our experiments performed at strain rates of ≈10^−2^. The high stresses associated with the experiments suggest that Coble creep might compete with other creep processes that would be anticipated to be more facile at these stresses and temperatures. Coble creep, however, would be necessary to facilitate long-range transport to the ZrO_2_ surface. The presence of a thin oxidation layer on the tungsten is hypothesized to induce this deformation mode and drive the nucleation of tungsten away from this surface oxidation product, as will be discussed further below. It should also be noted that similar long-range diffusion appears to be active during compression of the sample tested at 850 °C, although to a lesser extent. In that case, tungsten contamination pre-existed on the ZrO_2_ surface adjacent to the pillar. It may be seen that an adjacent tungsten metal particle on the ZrO_2_ surface grows during pillar compression, likely from long range diffusion away from the pillar.

The sample tested at 1250 °C was not dimensionally stable upon application of load as shown in Figure 3c. The pillar appears to retract while an apparent reaction product evolves between the sample and the indenter at a stress that is effectively zero. We hypothesize that the initial dimensional stability of the pillar is provided by an oxidation layer, which can break up under an applied load and result in further reaction. Although the indenter continues to displace toward the reaction product and interact with it, after an initial load drop associated with deformation of the pillar, the stress is not measurable upon further displacement. Weak interactions with the indenter, however, clearly affect the reaction pathway of the former pillar.

Additional pillars were tested at lower temperatures after in situ pre-annealing at high temperatures > 1000 °C. Surface oxidation was observed during FIB preparation and care was taken to avoid the inclusion of bulk oxide in the samples, although it is possible that high levels of dissolved oxygen and oxide at defects such as grain boundaries could be present. Those nanopillar samples produced yield strength values of 0.35 GPa at room temperature. This value is comparable to reports of yield stress in bulk materials, but is low relative to single crystal nanopillars [13]. These results suggest that the increase in grain size could account for much of the decrease in yield strength above 660 °C.

## 4. Discussion

**Figure 4:**
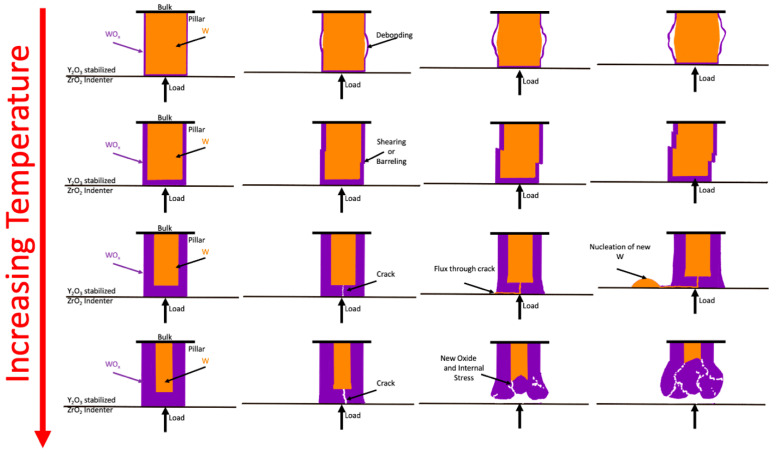
Schematic of the hypothesized deformation response observed in our experiments different temperature ranges. At lower temperatures, the thinner oxidation layer is hypothesized to have limited effect on the deformation modes. At higher temperatures, the constraint of a thicker oxidation layer is hypothesized to drive localized plasticity via a creep mechanism. Rapid oxidation occurs at 1250 °C.

Figure 4 schematically depicts the proposed evolution of the samples during mechanical testing at different temperatures. As temperature increases, the effect of surface oxidations becomes an increasingly important factor in determining the deformation modes active in the pillars. At low temperatures, the thin oxidation scales are hypothesized to only weakly affect mechanical properties and deformation modes. For example, the decohesion of the thin scale observed at 285 °C appears to have little impact on the mechanical properties. At higher temperatures, the constraint of the oxidation product appears to drive long-range diffusional transport in response to the applied load, i.e., creep. For example, deformation at 1120 °C occurred via the removal of tungsten from the pillar and its redeposition on the YSZ indenter. The relatively uniform pillar deformation and the formation of relatively spherical deposits suggest the process is diffusional in nature. The delocalized nature of the deformation process is hypothesized to result from the presence of a surface oxidation product. The presence of an oxidation product is visible in ex situ SEM characterization of the pillars. The composition of this surface reaction product was not determined directly. Related experiments performed in this same microscope at elevated temperatures, for example, have characterized the oxidation of metals in detail [27]. Common vacuum impurities, H_2_, CO, CO_2_, O_2_, N_2_, H_2_O, and hydrocarbons, are most likely to form tungsten oxides, nitrides, or carbides, given the relative instability of tungsten carbontes. Tungsten carbides and nitrides, however, are susceptible to oxidation are relatively low oxygen partial pressures. The mechanism associated with long range mass transport is proposed to be analogous to whisker growth mechanisms that are relevant to the formation of both whiskers and nodular features in alloys [35,36].

The whisker growth mechanism remained elusive for over half a century in electronic devices, whose function it has impacted since the earliest vacuum tube–based systems [37]. Whisker growth is commonly observed in low melting point metals used in room temperature applications, such as Sn-based solder, Li dendrite growth during electrodeposition, Zn galvanic coatings, and cadmium, lead, bismuth, and silver for electronics applications [35,36,38,39,40]. Observations of tungsten whisker growth have been reported in early studies of the process [41]. The effect was attributed to possible vapor-phase transport, but the whisker growth mechanism was not well understood at that time. The whisker growth mechanism requires three conditions: (1) a compressive stress state, (2) a surface oxidation layer that constrains uniform stress relaxation processes, and (3) facile diffusion that mediates creep [35,36,42]. Regions of the oxidation scale that are relatively weak can fracture, enabling mass flux to the low stress region that results in highly localized plasticity. This flux results in whisker growth to lengths on the order of microns or even millimeters, and exhibit morphologies varying between relatively equiaxed nodules and hillocks to long, wire-like features [35,38,43]. Several studies of tungsten fuzz have reported the presence of surface oxide, satisfying Condition (1). Oxygen impurities are observed in fusion plasmas at concentrations larger than are thermodynamically necessary to oxidize tungsten [44]. Oxygen has been demonstrated to be present on tungsten fuzz surfaces as determined by energy dispersive spectroscopy and X-ray photoelectron spectroscopy [1,3,45]. In electronics applications, residual stress from thermal processing or thermal cycling during operation drive whisker growth from the surface [46]. For plasma-facing materials, gas implantation induces a large compressive stress within the tungsten bulk as well as the growing fuzz, satisfying Condition (2). The stress is large enough to induce bubble growth and should drive whisker growth if Condition (3) is met. Our experiments suggest that tungsten is susceptible to the whisker growth at temperatures relevant to their application in plasma-facing environments. Coble creep is, furthermore, anticipated to be active in this temperature range at these length-scales [11]. It is, thus, hypothesized that a whisker-type mechanism could contribute, at least in part, to the evolution of tungsten fuzz.

Whiskers and fuzz share many common morphological features. There are, however, a number of differences related to microstructural length scales and the presence of internal porosity resulting from He implantation. While the latter difference is simple to rationalize, the former is less trivial. Whiskers have been observed to emerge from fractures in the surface oxide, which can, in some cases, define the length-scale and surface topography of the whisker [47]. It has been observed at the early stages of fuzz growth that tungsten can penetrate an amorphous oxide layer without fracturing it, presumably through a creep process [48]. Distinctions between the processes that give rise to initiation of the localized plasticity could account for morphological differences if the whisker growth process indeed underlies fuzz growth. The complexities of plasmas’ environments mean fuzz growth should likely be more complex than whisker growth. Fuzz growth includes the evolution of internal bubbles, irradiation-induced point and line defects, and the motion of those defects should impact the evolution of fuzz during irradiation. These relevant effects have already been discussed in the context of existing models for fuzz growth [6,7,8]. Nevertheless, the observations of surface oxide in tungsten fuzz evolving systems, along with our current results indicating that whisker growth can be active in the fuzz growth temperature regime in tungsten, suggest that the whisker growth mechanism can contribute to fuzz growth. It is noted that the current work only raises the possibility that these two mechanisms may be related. Related high temperature small scale nanomechanical testing under ion irradiation [25] using samples of model geometry are planned to more definitively clarify the relationship between these two mechanisms.

## 5. Conclusions

Nanopillar compression experiments were performed as a function of temperature up to 1250 °C using a novel method based on laser heating of a metallic film on a thermally insulating substrate. The low temperature deformation is reasonably consistent with prior room temperature experiments. Debonding of a surface scale is observed between 200 °C and 285 °C, and, by 1250 °C, the pillars fully react upon application of load. At temperatures where creep is active in tungsten, pillar compression drives mass from the pillar into the formation and growth of particles on the indenter surface. The presence of the surface scale is hypothesized to account for this localization of deformation in a manner consistent with the whisker growth mechanism. The results are consistent with prior observations of whisker growth in tungsten, but suggest that such features could evolve via plasticity rather than a vapor transport mechanism. This result is of contemporary practical interest, because it is hypothesized that the conditions for fuzz growth meet those necessary to induce whisker growth. Given the susceptibility of tungsten to the whisker growth process at temperatures and oxygen partial pressures relevant to fuzz growth, it is hypothesized that this mechanism may contribute to fuzz growth.

## Data Availability

The data is available on reasonable request from the corresponding author.

## References

[1] Baldwin M.J., Doerner R.P. (2010). Formation of helium induced nanostructure ‘fuzz’ on various tungsten grades. J. Nucl. Mater..

[2] Kajita S., Sakaguchi W., Ohno N., Yoshida N., Saeki T. (2009). Formation process of tungsten nanostructure by the exposure to helium plasma under fusion relevant plasma conditions. Nucl. Fusion.

[3] Wang K., Doerner R.P., Baldwin M.J., Meyer F.W., Bannister M.E., Darbal A., Stroud R., Parish C.M. (2017). Morphologies of tungsten nanotendrils grown under helium exposure. Sci. Rep..

[4] Kajita S., Yoshida N., Ohno N. (2020). Tungsten fuzz: Deposition effects and influence to fusion devices. Nucl. Mater. Energy.

[5] Woller K.B., Whyte D.G., Wright G.M. (2017). Isolated nano-tendril bundles on tungsten surfaces exposed to radiofrequency helium plasma. Nucl. Mater. Energy.

[6] Krasheninnikov S.I. (2011). Viscoelastic model of tungsten ‘fuzz’ growth. Phys. Scr..

[7] Lasa A., Tähtinen S.K., Nordlund K. (2014). Loop punching and bubble rupture causing surface roughening—A model for W fuzz growth. EPL (Europhys. Lett.).

[8] Miyamoto M., Watanabe T., Nagashima H., Nishijima D., Doerner R.P., Krasheninnikov S.I., Sagara A., Yoshida N. (2014). In situ transmission electron microscope observation of the formation of fuzzy structures on tungsten. Phys. Scr..

[9] Martynenko Y.V., Nagel’ M.Y. (2012). Model of fuzz formation on a tungsten surface. Plasma Phys. Rep..

[10] Ashby M.F. (1974). A first report on sintering diagrams. Acta Met..

[11] Webb J., Gollapudi S., Charit I. (2019). An overview of creep in tungsten and its alloys. Int. J. Refract. Met. Hard Mater..

[12] Lee S.-W., Cheng Y., Ryu I., Greer J.R. (2014). Cold-temperature deformation of nano-sized tungsten and niobium as revealed by in-situ nano-mechanical experiments. Sci. China Technol. Sci..

[13] Kim J.-Y., Jang D., Greer J.R. (2010). Tensile and compressive behavior of tungsten, molybdenum, tantalum and niobium at the nanoscale. Acta Mater..

[14] Smirnov R.D., Krasheninnikov S.I. (2013). On the shear strength of tungsten nano-structures with embedded helium. Nucl. Fusion.

[15] Zhu F., Wang D., Gao N., Peng H., Xie Z., Zhang Z. (2019). Microstructure evolution and Young′s modulus of He-implanted nanocrystalline tungsten film. J. Nucl. Mater..

[16] Vikrant K.S.N., Grosso R.L., Feng L., Muccillo E.N.S., Muche D.N.F., Jawaharram G.S., Barr C.M., Monterrosa A.M., Castro R.H.R., García R.E. (2020). Ultrahigh temperature in situ transmission electron microscopy based bicrystal coble creep in zirconia I: Nanowire growth and interfacial diffusivity. Acta Mater..

[17] Sharma A., Gazit N., Klinger L., Rabkin E. (2019). Pseudoelasticity of Metal Nanoparticles Is Caused by Their Ultrahigh Strength. Adv. Funct. Mater..

[18] Dehm G., Jaya B.N., Raghavan R., Kirchlechner C. (2018). Overview on micro- and nanomechanical testing: New insights in interface plasticity and fracture at small length scales. Acta Mater..

[19] Tan E.P.S., Lim C.T. (2006). Mechanical characterization of nanofibers—A review. Compos. Sci. Technol..

[20] Greer J.R., Kim J.-Y., Burek M.J. (2009). The in-situ mechanical testing of nanoscale single-crystalline nanopillars. JOM.

[21] Cho J., Li Q., Wang H., Fan Z., Li J., Xue S., Vikrant K.S.N., Wang H., Holland T.B., Mukherjee A.K. (2018). High temperature deformability of ductile flash-sintered ceramics via in-situ compression. Nat. Commun..

[22] Grosso R.L., Muccillo E.N.S., Muche D.N.F., Jawaharram G.S., Barr C.M., Monterrosa A.M., Castro R.H.R., Hattar K., Dillon S.J. (2020). In Situ Transmission Electron Microscopy for Ultrahigh Temperature Mechanical Testing of ZrO2. Nano Lett..

[23] Niekiel F., Kraschewski S.M., Müller J., Butz B., Spiecker E. (2017). Local temperature measurement in TEM by parallel beam electron diffraction. Ultramicroscopy.

[24] Grosso R.L., Vikrant K.S.N., Feng L., Muccillo E.N.S., Muche D.N.F., Jawaharram G.S., Barr C.M., Monterrosa A.M., Castro R.H.R., Garcia R.E. (2020). Ultrahigh temperature in situ transmission electron microscopy based bicrystal coble creep in Zirconia II: Interfacial thermodynamics and transport mechanisms. Acta Mater..

[25] Jawaharram G.S., Barr C.M., Monterrosa A.M., Hattar K., Averback R.S., Dillon S.J. (2019). Irradiation induced creep in nanocrystalline high entropy alloys. Acta Mater..

[26] Miiller A.P., Cezairliyan A. (1990). Thermal expansion of tungsten in the range 1500?3600 K by a transient interferometric technique. Int. J. Thermophys..

[27] Donaldson O.K., Hattar K., Trelewicz J.R. (2016). Metastable Tantalum Oxide Formation During the Devitrification of Amorphous Tantalum Thin Films. J. Am. Ceram. Soc..

[28] Blackburn P.E., Hoch M., Johnston H.L. (1958). The Vaporization of Molybdenum and Tungsten Oxides. J. Phys. Chem..

[29] Ackermann R.J., Rauh E.G. (1963). A Thermodynamic Study of the Tungsten-Oxygen System at High Temperatures1. J. Phys. Chem..

[30] Sun L., Noh K.W., Wen J.-G., Dillon S.J. (2011). In Situ Transmission Electron Microscopy Observation of Silver Oxidation in Ionized/Atomic Gas. Langmuir.

[31] Velicu I.-L., Tiron V., Porosnicu C., Burducea I., Lupu N., Stoian G., Popa G., Munteanu D. (2017). Enhanced properties of tungsten thin films deposited with a novel HiPIMS approach. Appl. Surf. Sci..

[32] Sun H.L., Song Z.X., Guo D.G., Ma F., Xu K.W. (2010). Microstructure and Mechanical Properties of Nanocrystalline Tungsten Thin Films. J. Mater. Sci. Technol..

[33] Zhang P., Li S.X., Zhang Z.F. (2011). General relationship between strength and hardness. Mater. Sci. Eng. A.

[34] Donaldson O.K., Hattar K., Kaub T., Thompson G.B., Trelewicz J.R. (2017). Solute stabilization of nanocrystalline tungsten against abnormal grain growth. J. Mater. Res..

[35] Chason E., Jadhav N., Pei F., Buchovecky E., Bower A. (2013). Growth of whiskers from Sn surfaces: Driving forces and growth mechanisms. Prog. Surf. Sci..

[36] Lindborg U. (1976). A model for the spontaneous growth of zinc, cadmium and tin whiskers. Acta Met..

[37] Zhang P., Zhang Y., Sun Z. (2015). Spontaneous Growth of Metal Whiskers on Surfaces of Solids: A Review. J. Mater. Sci. Technol..

[38] Wu L., Ashworth M.A., Wilcox G.D. (2015). Zinc whisker growth from electroplated finishes—A review. Trans. IMF.

[39] Xian A.-P., Liu M. (2012). Effect of humidity on tin whisker growth from Sn3Nd intermetallic compound. J. Mater. Res..

[40] Illés B., Krammer O., Hurtony T., Dušek K., Bušek D., Skwarek A. (2020). Kinetics of Sn whisker growth from Sn thin-films on Cu substrate. J. Mater. Sci. Mater. Electron..

[41] Dunn B. (1976). Whisker Formation on Electronic Materials. Circuit World.

[42] Sarobol P., Blendell J.E., Handwerker C.A. (2013). Whisker and hillock growth via coupled localized Coble creep, grain boundary sliding, and shear induced grain boundary migration. Acta Mater..

[43] Chen W.-H., Wang C., Sarobol P., Blendell J., Handwerker C. (2020). Local variations in grain formation, grain boundary sliding, and whisker growth along grain boundaries in large-grain Sn films. Scr. Mater..

[44] Bhattacharya A., Ghosh J., Chowdhuri M.B., Munshi P. (2020). Numerical estimation of the oxygen impurity transport in the Aditya tokamak. Phys. Plasmas.

[45] Kovach Y.E., Zhang F., Gao F., Foster J.E. (2019). Study of plasma induced nanostructure formation and surface morphology changes on tungsten and stainless steel at atmospheric pressure. J. Vac. Sci. Technol. A.

[46] Wang Y., Blendell J.E., Handwerker C. (2013). Evolution of tin whiskers and subsiding grains in thermal cycling. J. Mater. Sci..

[47] Jagtap P., Kumar P. (2020). Whisker Growth in Sn Coatings: A Review of Current Status and Future Prospects. J. Electron. Mater..

[48] El-Atwani O., Gonderman S., Suslov S., Efe M., De Temmerman G., Morgan T., Bystrov K., Hattar K., Allain J.P. (2015). Early stage damage of ultrafine-grained tungsten materials exposed to low energy helium ion irradiation. Fusion Eng. Des..

